# Exacerbation model of cumulative adverse experiences: prevalence, characteristics, and risk factors of self-harm and suicidality among Chinese migrant workers

**DOI:** 10.3389/fpubh.2025.1594466

**Published:** 2025-06-18

**Authors:** Tang Jiayi, Mengting Wang, Juan Fang, Wenqian Jian, Hong Pan, Xinyu Hu, Yanlong Liu, Li Chen, Linhui Liu

**Affiliations:** ^1^School of Mental Health, Wenzhou Medical University, Wenzhou, China; ^2^Student Affairs Office, South China Normal University, Guangzhou, China; ^3^The Affiliated Wenzhou Kangning Hospital, Wenzhou Medical University, Wenzhou, China; ^4^Lishui Key Laboratory of Mental Health and Brain Disorders, Lishui Second People’s Hospital, Lishui, China

**Keywords:** suicide, self-harm, cumulative risk, adverse childhood experiences, migrants

## Abstract

**Background:**

Self-harm and suicidality represent critical public health issues, particularly among migrant workers in China, who often confront adverse living and working conditions. This study aimed to investigate the prevalence and characteristics of self-harm and suicidality, explore the risk factors associated with adverse childhood experiences (ACEs) and adverse adulthood experiences (AAEs), and elucidate the relationship models between cumulative risk factors and self-harm and suicidality among Chinese migrant workers.

**Methods:**

We conducted a cross-sectional survey involving 2,739 rural-to-urban migrant workers across China. Participants completed a structured questionnaire assessing self-harm, suicidality, ACEs, AAEs, and sociodemographic characteristics. Data analysis involved descriptive statistics, cross-tabulation, independent samples t-tests, logistic regression, and stepwise regression.

**Results:**

Among the participants, the prevalence of self-harm and suicidality was 12.6 and 10.4%, respectively. Both ACEs and AAEs showed significant associations with self-harm and suicidality. Individuals reporting ACEs, such as parental divorce, childhood exposure to community violence, and school dropout, as well as AAEs including adult poverty, divorce intention, parent–child conflict, work burnout, and workplace discrimination, exhibited increased tendencies toward self-harm and suicidality. Moreover, a significant positive correlation was found between the cumulative risk index and self-harm and suicidality among Chinese migrant workers, with a critical threshold identified at 4–5 risk factors, indicating an exacerbation model.

**Conclusion:**

This study underscores the high prevalence of self-harm and suicidality among Chinese migrant workers, highlighting the significant impact of cumulative ACEs and AAEs on these outcomes. The findings emphasize the necessity for targeted interventions that address the identified risk factors to enhance the mental health and well-being of this vulnerable population.

## 1 Introduction

Self-harm and suicidality represent significant public health challenges, particularly among Chinese migrant workers, who often face adverse living and working conditions (1, 2). The migrant worker population in China is substantial, comprising 295.6 million rural migrant workers, which account for over one-third of the workforce in 2022 (3). Despite their significant contributions to urban productivity and GDP, these workers are vulnerable to substandard housing, financial instability, social exclusion, and inadequate access to mental health services (4–8). These factors collectively elevate their susceptibility to engaging in risky health behaviors such as self-harm and suicidality (9, 10). Previous research has highlighted elevated rates of self-harm and suicidality among this population, with a lifetime prevalence of suicidal ideation reaching 12.8% (11) and self-harm rates ranging from 36 to 66.7% among young migrants (10). These findings underscore the urgent need to understand the underlying risk factors and implement targeted interventions to address these behaviors in this population.

Adverse childhood experiences (ACEs) and adverse adulthood experiences (AAEs) are well-documented risk factors for self-harm and suicidality (12). ACEs, including physical, sexual, and emotional abuse, neglect, and household dysfunction during childhood (13), have been shown to increase the likelihood of mental health issues, substance abuse, and suicidal behaviors (14–16). In China, a notable proportion of rural–urban migrant workers come from migrant family backgrounds, making them particularly vulnerable to ACEs. This vulnerability arises from factors such as parental separation, inconsistent caregiving, financial difficulties, social exclusion, and limited access to support services (4). A recent study by Li et al. found a significant association between ACEs and an increased likelihood of suicidal ideation among Chinese migrant workers (17). Similarly, AAEs, including poverty, unemployment, work burnout, and social discrimination further exacerbate the risk of self-harm and suicidality (18, 19). However, the cumulative impact of these adverse experiences on self-harm and suicidality among Chinese migrant workers remains under explored.

The cumulative risk model posits that the accumulation of multiple risk factors over time leads to widening disparities in health outcomes (20). This model suggests that individuals exposed to multiple adverse experiences are at a heightened risk of developing mental health issues and engaging in self-harm or suicidal behaviors (21–23). Existing research has demonstrated a dose–response relationship between the number of ACEs and negative health outcomes (24, 25), but little is known about how ACEs and AAEs interact to influence self-harm and suicidality among Chinese migrant workers. Additionally, Rauer et al. (26) identified three relationship models between cumulative risk and problem behavior: (1) the linear model, where multiple risk factors combine to influence outcomes, with minimal impact from a single factor; (2) the exacerbation model, in which the impact of cumulative risks rapidly escalates beyond a critical threshold, diminishing intervention effectiveness; and (3) the saturation model, where the effect of additional risk factors decreases after a critical point, indicating optimal timing for intervention. To date, no study has systematically examined these relationship models in the context of cumulative ACEs and AAEs in relation to self-harm or suicidality among Chinese migrant workers.

This study employs the cumulative risk model to examine the combined effects of adverse experiences in both childhood and adulthood on self-harm and suicidality among migrant workers. The objectives of this research are to (1) investigate the prevalence and characteristics of self-harm and suicidality, (2) explore and validate the risk factors of ACEs and AAEs for self-harm and suicidality among Chinese migrant workers, (3) identify the relationship models between cumulative risk factors and self-harm and suicidality.

## 2 Methods

### 2.1 Participants

This study recruited 2,891 rural-to-urban migrant workers from the Yangtze River Delta region, which is located in a plain where the Yangtze River flows into the East China Sea and is one of the largest economic production areas in China. The final eligible participants were selected using a stratified random sampling technique, consistent with our previous studies.

In Stage 1, three districts from four cities (Hangzhou, Ningbo, Wenzhou, and Jinhua) in the Yangtze River Delta region were randomly selected, representing inner-city, suburban, and urban fringe zones. In Stage 2, two residential sub-districts with a high density of migrant workers were randomly chosen from each of the three selected districts. In Stage 3, a quota-sampling procedure based on five occupational clusters was employed to ensure that the sample accurately represented the migrant worker population in China. These five clusters—manufacturing, construction, service industry, household services, and others—account for approximately 27.4, 18.7, 18.9, 19.2, and 15.8%, respectively, as reported in the “2020 Migrant Workers Monitoring Survey Report” released by the National Bureau of Statistics of China. Workplaces within these five clusters served as the sampling units, and random sampling was conducted within each stratum to select participants in proportion to the estimated size of that sector among migrant workers. In Stage 4, eligible participants were selected from the sampling units based on the following criteria: (1) possessing a rural hukou (household registration); (2) working in an urban area without holding a local hukou; and (3) being 18 years of age or older. Exclusion criteria included the inability to independently complete the survey, evident cognitive impairment, or symptoms of mental illness that could affect consent capacity or survey responses. Consequently, 2,891 migrant workers were deemed eligible for the study and consented to the study procedures, resulting in 2,739 valid responses and a response rate of 94.74%.

### 2.2 Procedure

A cross-sectional survey was conducted among rural-to-urban migrant workers using a structured questionnaire. The questionnaires were distributed online via computers, iPads, or smartphones. Initially, a pilot study was conducted with a pre-test involving 30 rural-to-urban migrant workers to assess the clarity, comprehensiveness, and acceptability of the questionnaire. Any duplicate, vague, or inappropriate questions were revised or removed. Subsequently, in the formal study, eligible respondents consented to and completed the online questionnaire via WeChat, the most popular social media platform in China. The questionnaires were administered by trained researchers, including faculty members and postgraduate students from Wenzhou Medical University, who had received systematic training prior to the formal study. The questionnaires were anonymous, and all participants participated in the study voluntarily.

### 2.3 Measurements

#### 2.3.1 Dependent variables

##### 2.3.1.1 Self-harm

Participants were surveyed on their self-harm behaviors in the past year through one specific question: “*In the past 12 months, did you ever bite, scratch, deliberately cut or burn yourself?*” Response choices were on a five-point Likert scale, and the response options were: 0 = “never,” 1 = “once a month,” 2 = “2 to 4 times a month,” 3 = “2 to 3 times a week,” and 4 = “4 times a week.” Higher scores indicate a greater degree of self-harm. Participants who answered “never” to all questions were categorized as having no self-harm, while those who responded otherwise were considered to exhibit self-harm.

##### 2.3.1.2 Suicidality

Suicidality comprises three elements: suicidal ideation, suicide planning, and suicide attempts (27). Drawing on prior studies (28–30), this research employed three inquiries to evaluate suicidality: “*Have you seriously considered suicide in the past year?*”; “*Have you planned how you would commit suicide in the past year?*”; and “*Have you taken steps to commit suicide in the past year?*”. All three questions were answered on a 5-point Likert scale, and the response options were: 0 = “never,” 1 = “once a month,” 2 = “2 to 4 times a month,” 3 = “2 to 3 times a week,” and 4 = “4 times a week.” Higher scores indicate a greater degree of suicidality. Participants who answered “*never*” to all questions were categorized as having no suicidality, while those who responded otherwise were considered to exhibit suicidality.

#### 2.3.2 Adverse childhood experiences

The present study utilized a 7-risk indicator in childhood. This included three factors within the family system, such as left-behind experience, childhood poverty, family violence, and divorced parents, as well as three factors within the school and social system, like school dropout, peer victimization, and community violence.

##### 2.3.2.1 Left-behind experience

A single question was used to gather information on it (“*did you ever had the left-behind experience before 16 years old?*”) (31) requiring respondents to choose between a “*yes*” or “*no*” answer.

##### 2.3.2.2 Childhood poverty

A single question from the Chinese version of Revised Adverse Childhood Experience Questionnaire (ACEQ-R) (32) was used to gather information on it. The question asked respondents, “*When you were a child, was your family in poor?*” with response options of “yes” or “no.”

##### 2.3.2.3 Family violence

A single question from the Chinese version of Revised Adverse Childhood Experience Questionnaire (ACEQ-R) (32) was used to gather information on it. The question asked respondents “*When you were a child, did your family ever beat you a lot?*” with response options of “yes” or “no.”

##### 2.3.2.4 Divorced parents

A single question from the Chinese version of Revised Adverse Childhood Experience Questionnaire (ACEQ-R) (32) was used to gather information on it. The question asked respondents, “*When you were a child, were your parents divorced?*” with response options of “yes” or “no.”

##### 2.3.2.5 School dropout

According to Liu et al. (33) and Wu et al. (34), information of school dropout of migrant workers during compulsory education was collected using a single question: “*Did you ever experience dropping out of school before the age of 16?*” Respondents were required to select either “yes” or “no” as their answer.

##### 2.3.2.6 Peer victimization

A single question from the Chinese version of Revised Adverse Childhood Experience Questionnaire (ACEQ-R) (32) was used to gather information on peer victimization. The question asked respondents, “*When you were a child, did you ever experience peer victimization?*” with response options of “yes” or “no”.

##### 2.3.2.7 Community violence

A single question from the Chinese version of Revised Adverse Childhood Experience Questionnaire (ACEQ-R) (32) was used to gather information on community violence. The question asked respondents, “*When you were a child, did you ever had the community violence experience?*” with response options of “yes” or “no.”

#### 2.3.3 Adverse adulthood experiences

##### 2.3.3.1 Care-giving to the older adult

It was assessed by a single question based on Železná (35): “*In the past 12 months, did you experience any difficulties in taking care of the older adult?*” Respondents were required to choose between a “yes” or “no” answer.

##### 2.3.3.2 Adulthood poverty

It was assessed by the Chinese version of Family Economic Hardship Scale (FEHS) (36, 37). The questionnaire consisted of eight questions, such as “*In the past 12 months, we did not have enough money for new clothes*,” which were rated on a 5-point Likert scale ranging from 0 = “never” to 4 = “always.” Higher scores on the questionnaire indicated a higher level of adulthood poverty. The FEHS has been found to have good reliability and validity in prior research, with Cronbach’s alpha coefficients typically ranging from 0.80 to 0.90. In this study, we used the 25th percentile as the criteria for defining the risk of poverty, categorizing individuals as either “yes” or “no” for being at risk.

##### 2.3.3.3 Divorce intention

The 5-items Chinese Marital Quality Scale (CMQS) (38) was used to assess divorce intention. In this scale, participants are asked to respond on a 4-point scale (the response options were from 1 = “never” to 4 = “recently”). Higher scores indicate a greater degree of divorce intention. Previous research demonstrates the CMQS has good internal consistency (Coefficient Alpha = 0.79). In this study, we conducted 25th percentile as the risk definition criteria, dichotomizing risk of poverty on “yes” or “no.”

##### 2.3.3.4 Parent–child conflict

The 12-item parent–child conflict subscale of the Parent- Child Relationship Scale (39) was used to assess parent–child conflict. In this scale, participants are asked to respond on a 5-point Likert scale (from 1 = “strongly disagree” to 5 = “strongly agree”). Higher scores indicate greater levels of parent–child conflict. Previous research demonstrates the parent–child conflict subscale has good internal consistency (Coeffcient Alpha = 0.84) (40). In this study, we conducted 25th percentile as the risk definition criteria, dichotomizing risk of poverty on “yes” or “no.”

##### 2.3.3.5 Work burnout

Work burnout was assessed using the Maslach Burnout Inventory (MBI) (41, 42), a 16-item self-report instrument that measures attitudes toward work on a 7-point Likert scale. Higher scores on the MBI indicate a higher level of work burnout. The MBI demonstrated good internal consistency across different countries with alpha values ranging from 0.85 to 0.89 (43, 44). In this study, the risk of work burnout was defined using the 25th percentile as the criteria, categorizing individuals into “yes” or “no” for the risk of work burnout.

##### 2.3.3.6 Work discrimination

The level of work discrimination was assessed using the revised 12-item Perceived Devaluation and Discrimination scale (PDDs) (45). Participants rated each question, such as “*Most people would like to make friends with migrant workers at work*,” on a 4-point Likert scale ranging from 1 = “strongly disagree” to 4 = “strongly agree.” A higher score indicated a greater level of perceived work discrimination. The internal consistency of the PDD scale has been found to be high, with Cronbach’s alpha coefficients typically ranging from 0.80 to 0.90, indicating good to excellent reliability. In this study, we defined the risk of work discrimination using the 25th percentile as the cutoff, categorizing individuals into “yes” or “no” for experiencing work discrimination.

#### 2.3.4 Covariates

Covariables included gender (binary, 0 for male and 1 for female) and education level (continuous, 1 for primary or below, 2 for junior high, 3 for senior high, 4 for college or above) and age (continuous) using self-administered questions.

#### 2.3.5 Quality control

The survey progress was closely monitored and the data accuracy was verified. Three quality control questions were incorporated to detect inattentive participants, who were asked to choose from “No/Strongly agree/Strongly disagree” options. Questionnaires containing more than two logical inconsistencies were disregarded. Furthermore, respondents who finished the survey in under 3 min were excluded to prevent “too fast” responses, as we deemed this the minimum time necessary for a valid survey completion.

### 2.4 Data analysis

The study presents the socio-demographic characteristics of the sample by detailing the frequency and percentage of each category. Various statistical analyses were conducted using SPSS 25.0, including descriptive statistics, cross-tabulation, independent samples t-test, logistic regression, and stepwise regression. The goodness of fit for the logistic regression model was evaluated using the Hosmer-Lemeshow test. Feature variables identified through logistic regression were utilized to create nomograms for self-harm and suicidality. Nomograms and Receiver Operating Characteristic (ROC) curves were generated using R software (version 4.3.2). The data were randomly divided into training and validation sets in a 7:3 ratio, with the training set employed for model development and the validation set for model evaluation. The discrimination ability of the nomograms was assessed using metrics such as the Area Under the Curve (AUC), calibration curve, and decision curve analysis (DCA). All statistical tests were two-tailed, with significance established at *p* < 0.05.

## 3 Results

### 3.1 Prevalence and characteristics of self-harm and suicidality of migrant workers

The data revealed that the average age of the participants was 40.33 years old (*SD* = 6.35), with a majority falling within the 30–50 years age range. Among the participants, 52.1% were females (*n* = 1,428) and 47.9% were males (*n* = 1,311). In terms of education, 16.4% (*n* = 449) had completed education up to primary school or below, 46.9% (*n* = 1,284) had junior high education, 23.9% (*n* = 654) had senior high education, and 12.9% (*n* = 352) reported having college-level education or higher.

Of these participants, 10.4% displayed some form of suicidality and 12.6% engaged in self-harm behaviors. Gender and education were significantly associated with self-harm (*p* < 0.01) but not with suicidality (*p* > 0.05). Specifically, males were more likely to exhibit self-harm behaviors (*χ*^2^ = 8.89*, p* < 0.01), and individuals with lower levels of education reported a higher prevalence of self-harm (*χ*^2^ *= 11.5, p < 0.01*). Age did not show a significant association with self-harm or suicidality (*p* > 0.05). factors from ACEs and AAEs demonstrated significant associations with self-harm and suicidality (*ps* < 0.001). More detailed demographic information can be found in Table 1.

**Table 1:** Demographic characteristics of self-harm and suicidality among the respondents (n = 2,739).

Variables	All participants	Self-harm			Suicidality		
	*N* = 2,739 (%)	No (*n* = 2,394)	Yes (*n* = 345)	*χ*^2^*/t*	No (*n* = 2,454)	Yes (*n* = 285)	*χ*^2^*/t*
Demographic factors							
Gender				8.89**			3.34
Female	1,428 (52.14%)	1,274 (89.2%)	154 (10.8%)		1,294 (90.6%)	134 (9.4%)	
Male	1,311 (47.86%)	1,120 (85.4%)	191 (14.6%)		1,160 (88.5%)	151 (11.5%)	
Education				11.05**			4.39
Primary or below	449 (16.39%)	385 (85.7%)	64 (14.3%)		404 (90.0%)	45 (10.0%)	
Junior high	1,281 (46.77%)	1,104 (86.0%)	180 (14.0%)		1,141 (88.9%)	143 (11.1%)	
Senior high	654 (23.88%)	582 (89.0%)	72 (11.0%)		583 (89.1%)	71 (10.9%)	
College or above	352 (12.85%)	323 (91.8%)	29 (8.2%)		326 (92.6%)	26 (7.4%)	
Age		40.35 ± 6.27	40.13 ± 6.87	0.57	40.40 ± 6.28	39.72 ± 6.84	1.60
ACEs							
Left-behind				13.24***			16.18***
No	2,310 (84.34%)	2,042 (88.4%)	268 (11.6%)		2,093 (90.6%)	217 (9.4%)	
Yes	429 (15.66%)	352 (82.1%)	77 (17.9%)		361 (84.1%)	68 (15.9%)	
Childhood poverty				13.24***			15.15***
No	1,802 (65.79%)	1,605 (89.5%)	197 (10.5%)		1,644 (91.2%)	158 (8.8%)	
Yes	937 (34.21%)	789 (84.2%)	148 (15.8%)		810 (86.4%)	127 (13.6%)	
Family violence				20.28***			29.56***
No	2,609 (95.25%)	2,297 (88.0%)	312 (12.0%)		2,356 (90.3%)	253 (9.7%)	
Yes	130 (4.75%)	97 (74.6%)	33 (25.4%)		98 (75.4%)	32 (24.6%)	
Divorced parents				6.21**			39.82***
No	2,625 (95.84%)	2,303 (87.7%)	322 (12.3%)		2,372 (90.4%)	253 (9.6%)	
Yes	114 (4.16%)	91 (79.8%)	23 (20.2%)		82 (71.9%)	32 (28.1%)	
School dropout				32.95***			10.83***
No	1,797 (65.61%)	1,618 (90.0%)	179 (10.0%)		1,635 (91.0%)	162 (9.0%)	
Yes	942 (34.39%)	776 (82.4%)	166 (17.6%)		819 (86.9%)	123 (13.1%)	
Peer victimization				15.94***			18.46***
No	2,564 (93.61%)	2,258 (88.1%)	306 (11.9%)		2,314 (90.2%)	250 (9.8%)	
Yes	175 (6.39%)	136 (77.7%)	39 (22.3%)		140 (80.0%)	35 (20.0%)	
Community violence				12.02***			9.88**
No	1,984 (72.44%)	1,761 (88.8%)	223 (11.2%)		1,800 (90.7%)	184 (9.3%)	
Yes	755 (27.56%)	633 (83.8%)	122 (16.2%)		654 (86.6%)	101 (13.4%)	
AAEs							
Care-giving to the older adult				14.06***			13.81***
No	1,635 (59.69%)	1,461 (89.4%)	174 (10.6%)		1,494 (91.4%)	141 (8.6%)	
Yes	1,104 (40.31%)	933 (84.5%)	171 (15.5%)		960 (87.0%)	144 (13.0%)	
Adulthood poverty				40.40***			57.06***
No	1,980 (72.29%)	1,780 (89.9%)	200 (10.1%)		1,828 (92.3%)	152 (7.7%)	
Yes	759 (27.71%)	614 (80.9%)	145 (19.1%)		626 (82.5%)	133 (17.5%)	
Divorce intention				71.78***			115.84***
No	2,009 (73.35%)	1,821 (90.6%)	188 (9.4%)		1,876 (93.4%)	133 (6.6%)	
Yes	730 (26.65%)	573 (78.5%)	157 (21.5%)		578 (79.2%)	152 (20.8%)	
Parent–child conflict				110.80***			106.15***
No	1,970 (71.92%)	1,804 (91.6%)	166 (8.4%)		1,839 (93.4%)	131 (6.6%)	
Yes	769 (28.08%)	590 (76.7%)	179 (23.3%)		615 (80.0%)	154 (20.0%)	
Work burnout				91.72***			112.28***
No	1,993 (72.76%)	1,816 (91.1%)	177 (8.9%)		1,861 (93.4%)	132 (6.6%)	
Yes	746 (27.24%)	578 (77.5%)	168 (22.5%)		593 (79.5%)	153 (20.5%)	
Work discrimination				66.53***			82.28***
No	1,893 (69.11%)	1,720 (90.9%)	173 (9.1%)		1,763 (93.1%)	130 (6.9%)	
Yes	846 (30.89%)	674 (79.7%)	172 (20.3%)		691 (81.7%)	155 (18.3%)	

*p-value < 0.05; **p-value < 0.01; ***p-value < 0.001. Bold values denote statistically significant improvements (*p* < 0.05).

### 3.2 Factors associated with self-harm and suicidality of migrant workers

#### 3.2.1 Risk predictors of self-harm and suicidality

In the unadjusted model, all factors stemming from ACEs or AAEs showed increased odds of self-harm and suicidality (refer to Table 2, located in the appendices following the reference list). When considering the fully adjusted model, individuals with divorced parents (AOR: 2.25, 95% CI: 1.38–3.65, *p* < 0.001) and exposure to community violence (AOR: 1.60, 95% CI: 1.21–2.12, *p* < 0.001) during childhood were more prone to engage in suicidality, even after accounting for gender, education, age, and other risk factors. Similarly, only school dropout (AOR: 1.31, 95% CI: 1.00–1.70, *p* < 0.05) and childhood exposure to community violence (AOR: 1.57, 95% CI: 1.21–2.03, *p* < 0.001) contributed to the odds of suicidality. Additionally, risk factors from AAEs remained robust as significant predictors of self-harm and suicidality, with the exception of care-giving difficulties for the older adult (*p* > 0.05). Through this phase of analysis, 7 key predictors of self-harm and suicidality were identified as statistically significant.

**Table 2:** Factors associated with self-harm and suicidality against rural-to-urban migrant workers (*n* = 2,739).

	Self-harm		Suicidality	
	COR (95%CI)	AOR (95%CI)	COR (95%CI)	AOR (95%CI)
ACEs				
Left-behind	1.67 (1.26–2.20)***	1.22 (0.90–1.67)	1.82 (1.35–2.44)***	1.19 (0.85–1.67)
Childhood poverty	1.53 (1.22–1.92)***	0.98 (0.74–1.29)	1.63 (1.27–2.09)***	1.00 (0.74–1.36)
Family violence	2.51 (1.66–7.78)***	1.42 (0.90–2.24)	3.04 (2.00–4.63)***	1.57 (0.98–2.52)
Divorced parents	1.81 (1.13–2.90)*	1.09 (0.65–1.84)	3.66 (2.38–5.62)***	2.25 (1.38–3.65)***
School dropout	1.93 (1.54–2.43)***	1.31 (1.00–1.70)*	1.52 (1.18–1.94)***	0.95 (0.70–1.27)
Peer victimization	2.12 (1.45–3.08)***	1.06 (0.69–1.63)	2.31 (1.56–3.43)***	0.95 (0.60–1.51)
Community violence	1.52 (1.20–1.93)***	1.57 (1.21–2.03)***	1.51 (1.17–1.96)**	1.60 (1.21–2.12)***
AAEs				
Care-giving to the older adult	1.54 (1.23–1.93)***	0.93 (0.71–1.22)	1.59 (1.24–2.03)***	0.89 (0.66–1.20)
Adulthood poverty	2.10 (1.67–2.65)***	1.32 (1.00–1.74)***	2.56 (1.99–3.28)***	1.52 (1.13–2.05)**
Divorce intention	2.65 (2.11–3.35)***	1.71 (1.32–2.21)***	3.71 (2.89–4.77)***	2.25 (1.70–2.98)***
Parent–child conflict	3.30 (2.62–4.15)***	2.02 (1.56–2.60)***	3.52 (2.74–4.52)***	1.91 (1.44–2.53)***
Work burnout	2.98 (2.37–3.76)***	1.59 (1.21–2.07)***	3.64 (2.83–4.67)***	1.81 (1.35–2.42)***
Work discrimination	2.54 (2.02–3.19)***	1.51 (1.17–1.96)**	3.04 (2.37–3.90)***	1.70 (1.28–2.26)***
**Cumulative risk groups (*n*)**	***	***	***	***
0 (*n* = 334)	1.00 (Reference)	1.00 (Reference)	1.00 (Reference)	1.00 (Reference)
1 (*n* = 469)	1.07 (0.54–2.14)	1.05 (0.53–2.11)	1.65 (0.67–4.06)	1.65 (0.67–4.07)
2 (*n* = 494)	2.07 (1.11–3.86)*	2.05 (1.10–3.83)*	3.24 (1.41–7.42)**	3.31 (1.44–7.60)**
3 (*n* = 416)	2.64 (1.42–4.91)**	2.54 (1.36–4.75)**	4.43 (1.94–10.08)***	4.53 (1.99–10.35)***
4 or 5 (*n* = 579)	4.49 (2.52–8.00)***	4.32 (2.41–7.74)***	7.06 (3.21–15.50)***	7.33 (3.33–16.15)***
6 or more (*n* = 447)	9.48 (5.35–16.80)***	8.99 (5.04–16.03)***	16.76 (7.70–36.46)***	17.19 (7.86–37.62)***

COR = Crude Odds Ratio. AOR = Adjusted Odds Ratio, represents the odds Ratio after adjusting for gender, education and age and other risk factors in the logistic regression model. CI, confidence interval. *p-value < 0.05; **p-value < 0.01; ***p-value < 0.001. Bold values denote statistically significant improvements (*p* < 0.05).

#### 3.2.2 Development of the predictive nomogram

**Figure 1:**
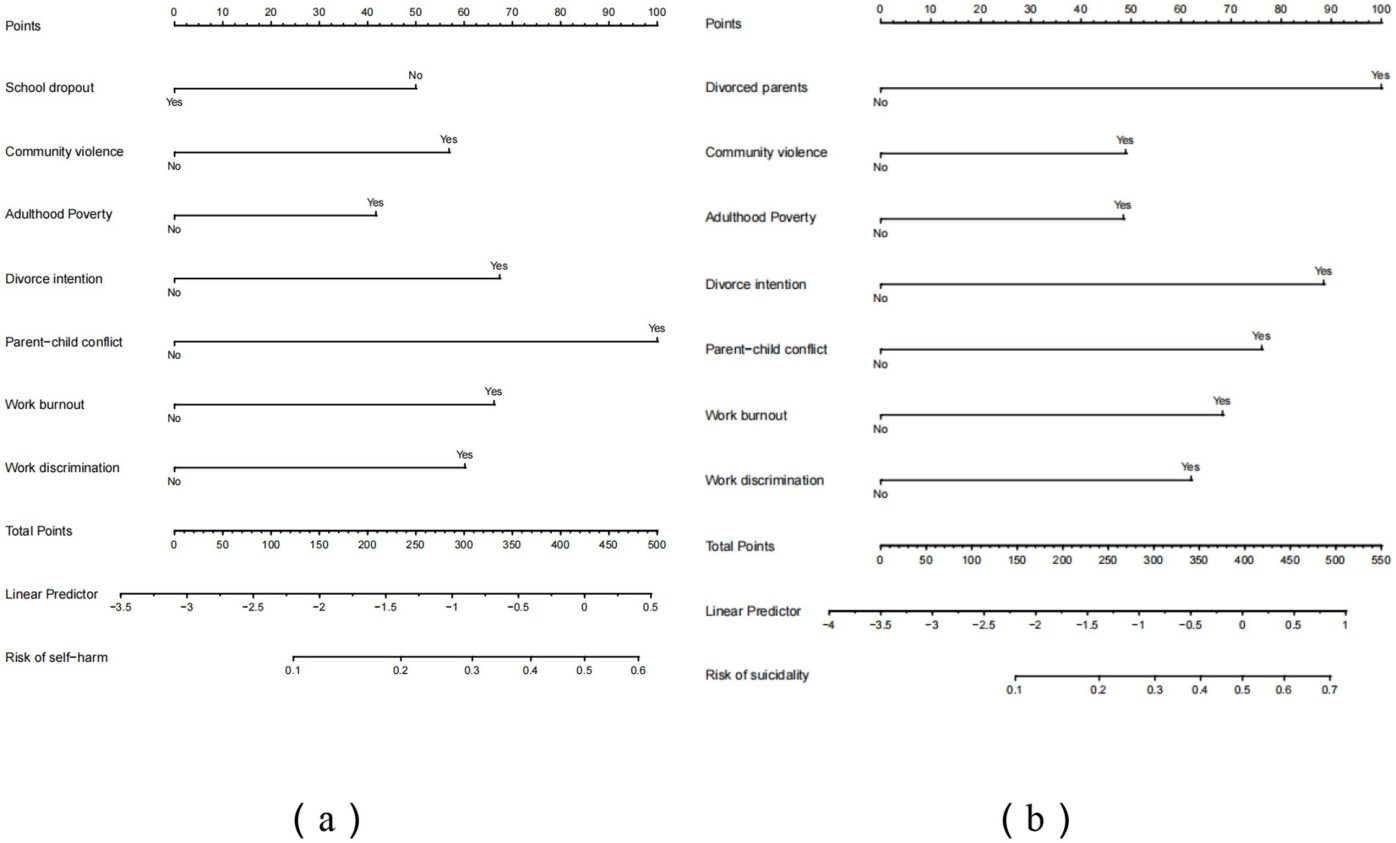
Proposed nomogram for self-harm **(a)** and suicidality **(b)**.

By analyzing the 7 optimal predictor variables of self-harm and suicidality, a nomogram was created, as depicted in Figures 1a,b. Each variable’s options were assigned corresponding scores, and the total score was calculated by summing the scores of all optimal predictor variables. The predictions of risks for different total scores are provided at the bottom of the figures. A higher total score indicates a greater probability of self-harm and suicidality. For example, if a man has a history of school dropout and exposure to community violence during childhood, along with intentions of divorce, risk of parent–child conflict, and work burnout (refer to Figure 1a), the scores for these variables would be approximately 50, 58, 68, 100, and 65, resulting in a total score of 341. According to the nomogram, this total score corresponds to an estimated probability of self-harm of around 32%. When he addes the risk of work discrimination, the scores for these 6 variables would be a total score of 401, estimating the risk of self-harm for around 80%.

#### 3.2.3 Performance and validation of the nomogram

**Figure 2:**
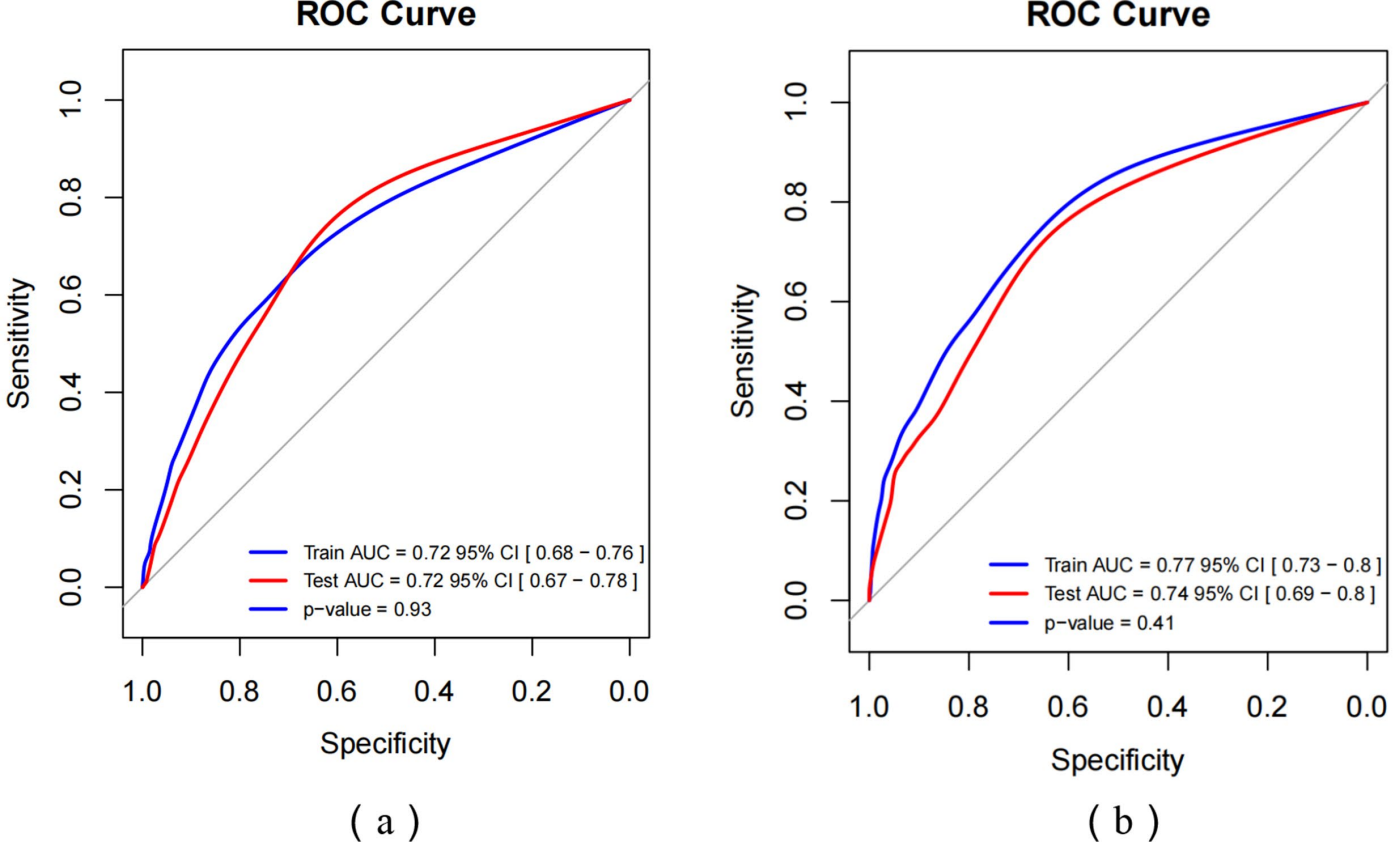
ROC curves of self-harm **(a)** and suicidality **(b)**.

**Figure 3:**
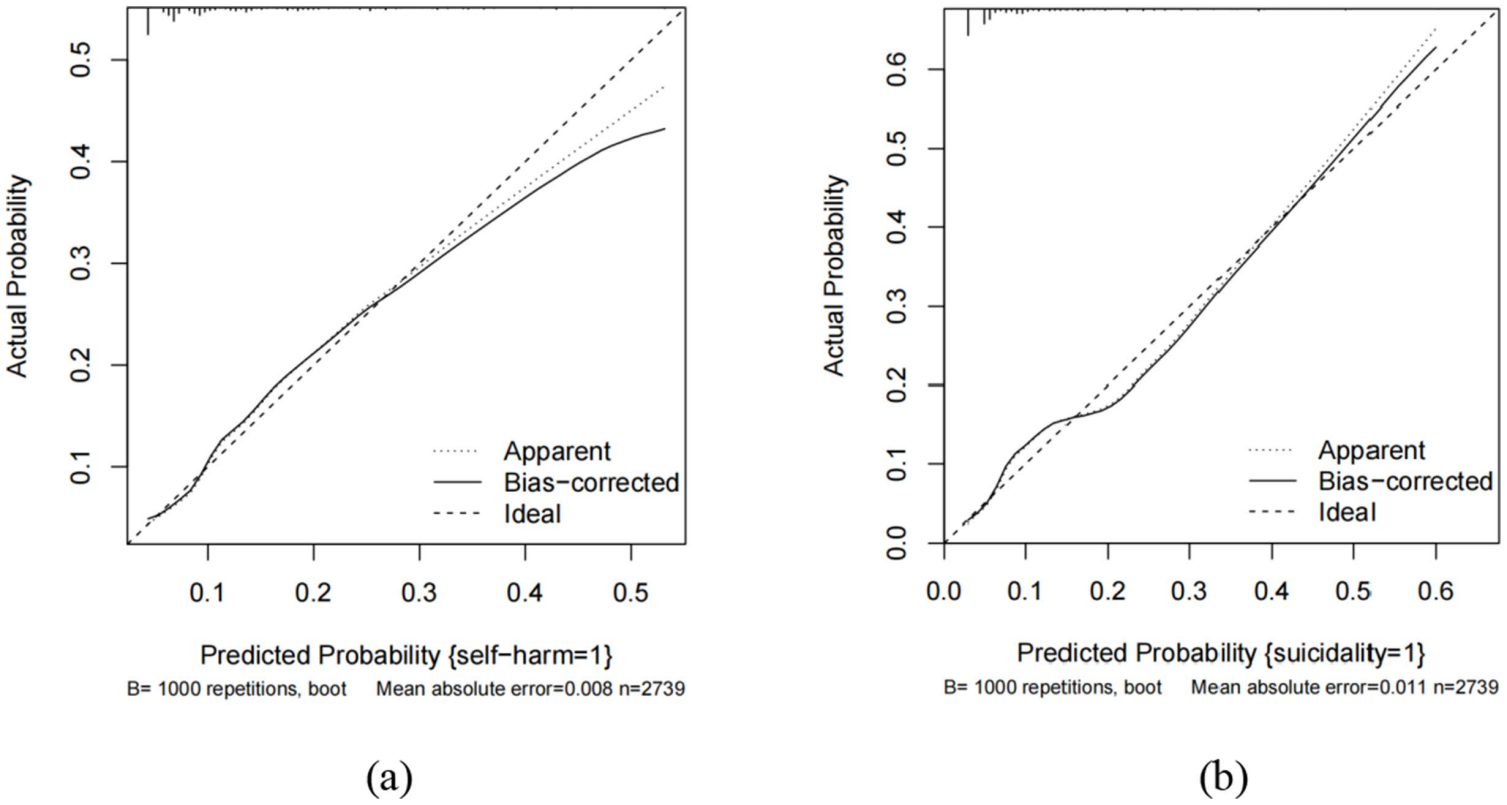
Calibration curves of the nomogram of self-harm **(a)** and suicidality **(b)** in the study.

**Figure 4:**
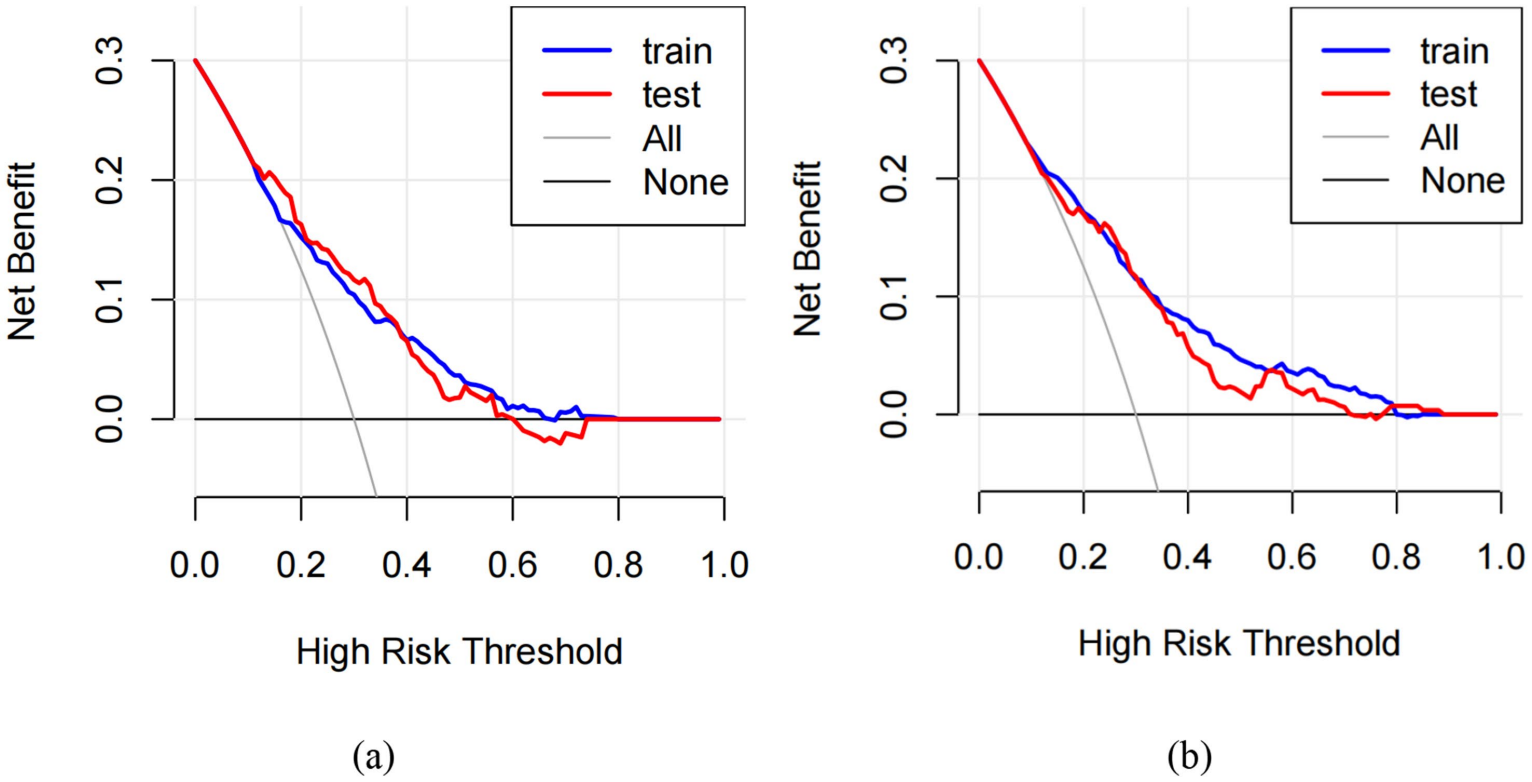
Decision curve analysis (DCA) for self-harm **(a)** and suicidality **(b)**.

ROC curves were used to assess the predictive value of key risk factors for self-harm and suicidality (see Figures 2a,b). The nomogram displayed an AUC of 0.72 (95% CI: 0.68–0.76) and 0.72 (95% CI: 0.67–0.78) for self-harm in both the training and validation sets. For suicidality, the AUC of training and validation sets was 0.72 (95% CI: 0.68–0.76) and 0.72 (95% CI: 0.67–0.78) for self-harm in both the training and validation sets 0.77 (95% CI: 0.73–0.80) and 0.74 (95% CI: 0.69–0.80). The values of sensitivity, specificity, Youden index and cutoff values for self-harm were 0.56, 0.79, 0.35, and 0.16, respectively. While for suicidality, they were 0.77, 0.63, 0.40, and 0.07. Calibration curves in both sets demonstrated good alignment between predicted and observed outcomes (refer to Figures 3a,b). The Hosmer–Lemeshow test confirmed the model’s adequacy (*p* > 0.05), and DCA indicated that the nomogram predictions provided greater net benefits at threshold probabilities of 12–59% _(a)_ and 13–71% _(b)_ (see Figures 4a,b). Overall, the nomograms developed in this study exhibited strong discrimination, calibration, clinical relevance, and potential for generalizability.

### 3.3 Association between cumulative risk index and self-harm and suicidality

**Figure 5:**
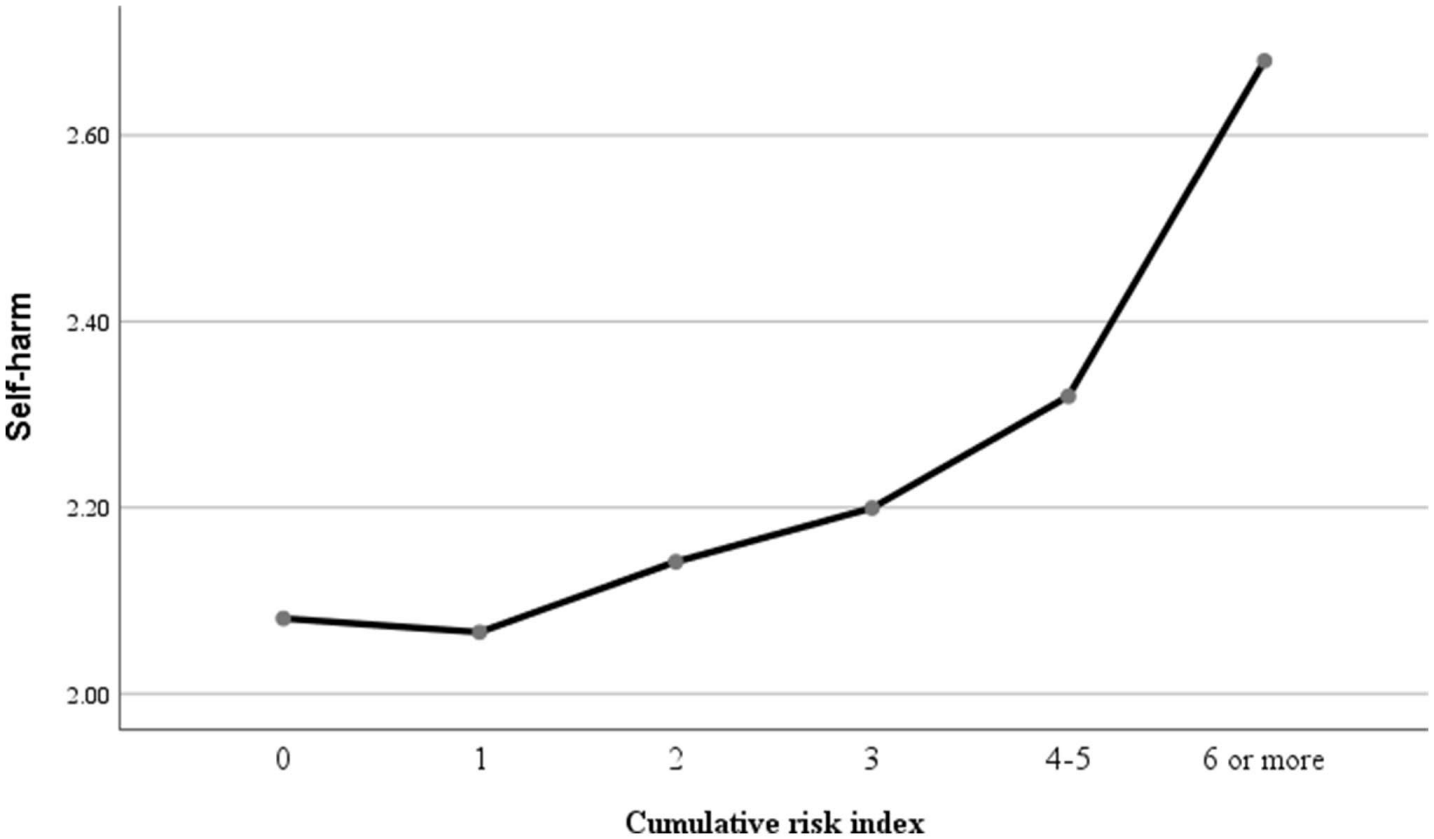
The relationship model between cumulative risk and self-harm.

**Figure 6:**
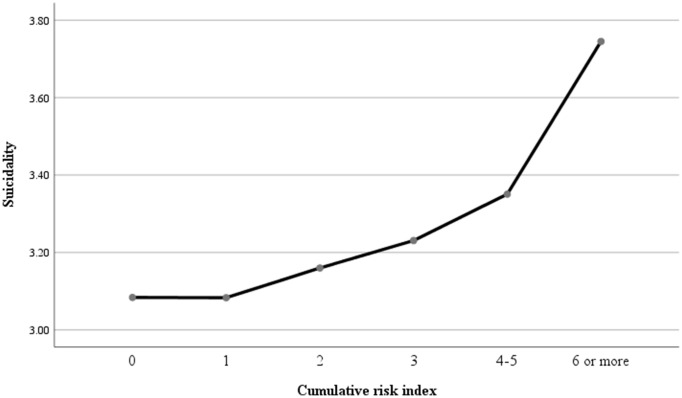
The relationship model between cumulative risk and suicidality.

To explore the impact of adversity on self-harm and suicidality, we computed a cumulative risk index for participants. Notably, we found no significant differences between cumulative risk index levels of 4 or 5 and self-harm (suicidality) (*p* > 0.05), therefore, we divided the participants into six groups. Specifically, the relationships models between cumulative risk index and self-harm (suicidality) were shown in Figures 5, 6. As shown in Table 2, when compared with those minor cumulative risk groups (0), participants with more cumulative risks tended to report more engagements of self-harm, with AORs of 1.05 (95% CI: 0.53–2.11, *p* > 0.05), 2.05 (95% CI: 1.10–3.83, *p <* 0.05), 2.54 (95% CI: 1.36–4.75, *p <* 0.01), 4.32 (95% CI: 2.41–7.74, *p <* 0.001) and 8.99 (95% CI: 5.04–16.03, *p <* 0.001) for 1, 2, 3, 4 (or 5), and 6 (or more) cumulative risk, respectively. Specifically, compared with 0 cumulative risks, the odds ratio of suicidality were 1.65 (95% CI: 0.67–4.07, *p* > 0.05), 3.31 (95% CI: 1.44–7.60, *p <* 0.01), 4.53 (95% CI: 1.99–10.35, *p <* 0.001), 7.33 (95% CI: 3.33–16.15, *p <* 0.001), and 17.19 (95% CI: 7.86–37.62, *p <* 0.001) for 1, 2, 3, 4 (or 5), and 6 (or more) cumulative risk, respectively.

Moreover, the study explored the relationship models between cumulative risk index and self-harm and suicidality. Initially, a bidirectional association was observed between cumulative risk index and self-harm as well as suicidality. The results indicated a significant positive correlation between cumulative risk index and self-harm (*r* = 0.23, *p* < 0.01) and suicidality (*r* = 0.21, *p* < 0.01). Subsequently, stepwise regression analysis was employed to assess the predictive power of cumulative risk on self-harm and suicidality, with gender, education, and age as control variables. The findings revealed that cumulative risk was a significant positive predictor of self-harm (β = 0.24, *p* = 0.001) and suicidality (β = 0.21, *p* = 0.001). Additionally, the T1 quadratic of cumulative risk index demonstrated a negative prediction for self-harm (β = 0.19, *p* < 0.001) and suicidality (β = 0.20, *p* < 0.001), suggesting an exacerbation model (see Table 3).

**Table 3:** Relationship between cumulative risk and self-harm and suicidality.

Variables	Self-harm						Suicidality					
	*R*^2^	*ΔR*^2^	*F(df)*	β	95% *CI*	β’	*R*^2^	*ΔR*^2^	*F(df)*	β	95% *CI*	β’
First step	0.009	0.009	8.06 (3)				0.004	0.004	4.06 (3)			
Gender				0.12	[0.05, 0.18]	0.07***				0.10	[0.02, 0.18]	0.05*
Education				−0.06	[−0.10, −0.002]	−0.07***				−0.06	[−0.11, −0.01]	−0.05**
Age				−0.007	[−0.01, −0.002]	−0.05**				−0.007	[−0.01, −0.001]	−0.04*
Second step	0.06	0.05	157.86 (1)				0.05	0.04	119.70 (1)			
Cumulative risk index				0.09	[0.07, 0.10]	0.24***				0.10	[0.08, 0.12]	0.21***
Third step	0.07	0.004	11.79 (1)				0.05	0.004	12.59 (1)			
The quadratic of cumulative risk index				0.008	[0.004, 0.013]	0.19***				0.01	[0.01, 0.02]	0.20***

β’ means the standardized β. *p-value < 0.05; **p-value < 0.01; ***p-value < 0.001.

## 4 Discussion

This study estimated the prevalence of self-harm and suicidality among Chinese rural-to-urban migrant workers and identified specific risk factors, including ACEs and AAEs, using the cumulative risk model. Predictive efficacy was assessed via a nomogram and ROC curves, providing novel insights into the quantitative impact of these risks. These findings represent the first exploration of cumulative risk in relation to self-harm and suicidality among Chinese rural-to-urban migrant workers, highlighting opportunities for targeted interventions.

### 4.1 Prevalence of self-harm and suicidality among Chinese migrant workers

In this study, the prevalence of self-harm and suicidality among Chinese migrant workers was found to be 12.6 and 10.4%, respectively. These findings align with previous research, which reported a lifetime prevalence of suicidal ideation among migrant workers at 12.8% (11) and a self-harm rate of 11.4% among young migrants (10). Notably, these rates are significantly higher than those reported in the general Chinese population. For instance, a meta-analysis of suicide rates in China indicated a lifetime prevalence of suicidal ideation of 3.9% and a 12-month prevalence of 2.2% (46). Similarly, a study among Chinese college students found a lifetime prevalence of self-harm at 6.4% (47). The heightened rates of self-harm and suicidality among migrant workers can be attributed to the unique challenges they encounter, including social isolation, discrimination, and limited access to mental health services. These factors exacerbate the psychological distress experienced by migrant workers, rendering them more vulnerable to self-harm and suicidality. The consistency of our findings with previous studies underscores the necessity for targeted interventions to address these behaviors within this vulnerable population.

### 4.2 Factors associated with self-harm and suicidality among Chinese migrant workers

Our study identified specific ACEs and AAEs that were significantly associated with an increased risk of self-harm and suicidality among Chinese migrant workers.

Specifically, childhood poverty, family violence, and peer victimization emerged as significant ACEs independently associated with elevated risk. These findings are consistent with existing literature demonstrating the enduring impact of adverse childhood experiences on mental well-being and subsequent risk for self-harm and suicidality (24, 48). For example, Abraham A. and Walker-Harding L. found that individuals exposed to domestic violence face significantly higher risks of substance abuse, repeated violence, and suicide in adulthood. This impact is especially profound for children from impoverished families, who have more than twice the risk of engaging in domestic violence as adults compared to other children (49). Additionally, experiences of peer victimization not only exacerbate immediate psychological distress but are also significantly linked to long-term self-harm behaviors. Adolescents who have been bullied have a 3.5 times higher risk of suicidal ideation compared to those who have not been bullied (50). Our findings are consistent with these studies, reinforcing the importance of ACEs as critical risk factors for self-harm and suicidality. The adverse childhood experiences appears to create a “toxic burden” that increases the likelihood of negative mental health outcomes in adulthood. However, the unique context of Chinese migrant workers, characterized by marginalization and limited access to mental health resources, may amplify the effects of these adverse experiences. Future interventions should focus on addressing ACEs early in life and providing support to mitigate their long-term impact.

Additionally, adulthood poverty, divorce intention, parent–child conflict, work burnout, and work discrimination were also identified as significant AAEs associated with self-harm and suicidality among Chinese migrant workers. These findings align with previous research and suggest that adverse experiences in adulthood can exacerbate mental health challenges and contribute to harmful behaviors within this population. Empirical studies have documented associations between adulthood poverty, work burnout, and work discrimination with increased rates of suicidal ideation, suicide attempts, and self-injury among Chinese adults and college students (2, 51). The unique challenges faced by Chinese migrant workers, including economic hardship and social exclusion, may exacerbate the effects of AAEs. For example, work burnout and discrimination can undermine an individual’s sense of self-worth and coping ability, while family conflicts may further isolate them from social support networks. Addressing these risk factors through targeted interventions, such as workplace mental health programs and family counseling, could help reduce the prevalence of self-harm and suicidality in this population.

Moreover, a key contribution of this study is the development of a nomogram designed to predict self-harm and suicidal behaviors among migrant workers. This innovative approach serves as a practical tool for identifying individuals at high risk, thereby facilitating early intervention and prevention efforts. The nomogram is constructed based on optimal predictive variables derived from logistic regression, providing a comprehensive framework for understanding the cumulative effects of ACEs and AAEs on these behaviors. This tool holds significant value for healthcare providers working with migrant workers, as it enables personalized risk assessments and tailored interventions.

### 4.3 Association between cumulative risk index and self-harm and suicidality

The findings of this study underscore the importance of considering the cumulative impact of ACEs and AAEs on self-harm and suicidality. Participants with a higher number of cumulative risks were significantly more likely to engage in these behaviors, demonstrating a clear positive correlation between the cumulative risk index and self-harm/suicidality. These results are consistent with the cumulative risk model, which posits that adverse experiences accumulate over time, leading to heightened risks of negative health outcomes (52).

Our study further suggests that the cumulative impact of ACEs and AAEs follows an exacerbation model, where the negative effects of adverse experiences do not simply add up but instead interact in ways that amplify their consequences (53). For instance, childhood poverty may hinder the development of effective coping mechanisms, making individuals more vulnerable to the negative impacts of poverty in adulthood (54). Similarly, exposure to family violence may impede the formation of healthy relationships, increasing susceptibility to parent–child conflict and other stressors (55, 56). Moreover, this exacerbation model may be especially pertinent for Chinese migrant workers, who may encounter distinct challenges and stressors that exacerbate the repercussions of adverse experiences. For example, discrimination and social exclusion faced by Chinese migrant workers can undermine their self-esteem and sense of belonging (2, 57). Additionally, obstacles to accessing healthcare and social services may impede their ability to cope with adverse experiences (58). These unique challenges and stressors may interact with adverse experiences to amplify their adverse effects on self-harm and suicidality.

Finally, the findings of this study indicate that the critical value in the exacerbation model is 4–5 risk factors, suggesting that the negative impact of adverse experiences on self-harm and suicidality may be more pronounced when individuals have encountered four or more adverse experiences. This aligns with a meta-analysis showing that individuals with at least four adverse experiences face an increased risk of various health outcomes (59, 60). The threshold effect model within the Cumulative Risk Model posits that the relationship between cumulative risk and self-harm/suicidality behaviors initially shows quantitative changes when the number of risk factors is below a certain threshold. However, once this critical threshold is reached, there is a notable shift in the association, leading to a significant increase in problematic behaviors with the addition of more risk factors. This underscores the importance of implementing effective interventions for self-harm and suicidality before reaching this critical threshold, as beyond this point, problem behaviors escalate in severity, posing greater challenges for intervention.

### 4.4 Strengths and limitations

This research has several important strengths. Firstly, the risk factors considered in the screening process, based on the cumulative risk theory, were both rigorous and comprehensive, encompassing a wide range of variables that significantly predicted self-harm and suicidality. Secondly, this study represents the first large-scale investigation focused on Chinese migrant workers, utilizing a nomogram to predict self-harm and suicidality behaviors. The nomograms and ROC curves provided valuable tools for identifying potential self-harm and suicidality behaviors among migrants, enabling timely intervention. Lastly, this paper quantitatively explores the relationship between the cumulative risk faced by migrant workers and their self-harm and suicidality behaviors, offering new empirical evidence on the link between cumulative risk and problem behaviors.

However, it is important to consider some limitations. One limitation is its cross-sectional design, which prevents establishing causal relationships between ACEs, AAEs, self-harm, and suicidality. While our findings suggest adverse experiences contribute to these behaviors, reverse causality cannot be ruled out. For example, individuals with pre-existing self-harm or suicidality tendencies may perceive or report their experiences differently, or their psychological state may influence how they interpret past and current adversities. Additionally, some adulthood risk factors, such as work burnout, discrimination, and parent–child conflict, may have bidirectional relationships with mental health outcomes. Longitudinal studies are needed to clarify temporal relationships, determine causality, and identify mediators and moderators. Another limitation is the use of self-report measures, which may be subject to recall bias and social desirability bias. Future studies could use more objective measures of ACEs and AAEs, such as administrative data or medical records.

### 4.5 Implication

This study emphasizes the urgent need for targeted interventions to address the high prevalence of self-harm and suicidality among Chinese migrant workers. The findings highlight the cumulative impact of ACEs and AAEs, with childhood poverty, family violence, peer victimization, adult poverty, work burnout, and workplace discrimination identified as key risk factors. The exacerbation model suggests that exceeding a critical threshold of these cumulative risks significantly increases the likelihood of self-harm and suicidality. This underscores the importance of multifaceted interventions addressing multiple risk factors simultaneously.

A key contribution of this study is the development of a nomogram to predict self-harm and suicidality behaviors among Chinese migrant workers. This evidence-based tool enables early identification of high-risk individuals and facilitates targeted prevention efforts. By integrating both ACEs and AAEs, the nomogram provides a comprehensive framework for personalized risk assessments, making it particularly valuable for healthcare providers working with this population.

Given the unique challenges faced by Chinese migrant workers, including economic hardship, social exclusion, and workplace discrimination, interventions must address these specific stressors. Workplace mental health programs, family counseling, and community-based support systems could mitigate the effects of AAEs such as work burnout and family conflict. Additionally, early intervention to address ACEs through education and social support could reduce their long-term impact on mental health.

Policymakers and healthcare providers should prioritize developing integrated strategies targeting both childhood and adulthood risk factors. For example, policies to reduce childhood poverty and family violence could have long-term mental health benefits, while workplace policies addressing burnout and discrimination could provide immediate relief. The nomogram could serve as a valuable tool to assess and monitor the effectiveness of these interventions.

## 5 Conclusion

In conclusion, this study provides compelling evidence of the high prevalence of self-harm and suicidality among Chinese migrant workers, with a strong emphasis on the cumulative impact of ACEs and AAEs as primary risk factors. By systematically analyzing the interplay of family, work, school, and social systems, the study establishes a clear link between the accumulation of risks and the heightened vulnerability of migrant workers to self-harm and suicidality behaviors. The findings underscore the necessity of comprehensive, integrated interventions to reduce the overall “toxic burden” of accumulated risk factors rather than focusing on isolated factors. For future interventions, early prevention of ACEs and workplace strategies to address AAEs, such as burnout and discrimination, are critical. The nomogram developed in this study provides a practical tool for identifying high-risk individuals and guiding targeted prevention efforts. These findings underscore the importance of comprehensive, evidence-based strategies to improve mental health outcomes for this vulnerable population.

## Data Availability

The datasets presented in this article are not readily available because all data used and/or analyzed in the present study are available from the corresponding author on reasonable request. They are not publicly available, in accordance with the Ethics Review Authority. Requests to access the datasets should be directed to Li Chen, psychologychenli@163.com.
